# Kinetics data from bovine sex-specific embryo development from three different bulls

**DOI:** 10.1016/j.dib.2016.03.066

**Published:** 2016-03-25

**Authors:** C.S. Oliveira, N.Z. Saraiva, M.R. de Lima, L.Z. Oliveira, R.V. Serapião, C.A.V. Borges, J.M. Garcia, L.S.A. Camargo

**Affiliations:** a Embrapa Dairy Cattle, Animal Reproduction Laboratory, Valença, Brazil; b Embrapa Amazônia Oriental, Belém, Brazil; c Sao Paulo State University, Jaboticabal, Brazil; d University of São Paulo (USP), Pirassununga, Brazil; e Centro Universitário de Rio Preto (UNIRP), São José do Rio Preto, Brazil; f PESAGRO-Rio, Animal Reproduction Laboratory, Valença, Brazil

## Abstract

Here we present kinetics data from bovine sex-specific embryo development. Embryos were originated using sex-sorted semen from three different Nelore bulls, and semen from the same batch was used for X-and Y-chromosome spermatozoa sorting. Data was obtained for six time points (24, 48, 96, 120, and 144 h.p.i.). Analyses for each bull׳s embryos (1, 2 and 3) is presented for female and male groups separately. Also, grouped data analysis, considering bull and sex interaction, is shown. For further interpretation and discussion, see "Cell death is involved in sexual dimorphism during preimplantation development" (Oliveira et al., 2015 [1]).

## Specifications Table

**Table:** 

Subject area	*Biology*
More specific subject area	*Embryonic Development*, *Reproductive Biology*, *IVF*
Type of data	*Table*, *graph*
How data was acquired	*Microscope* (*Olympus IX-*70)
Data format	*Analyzed*
Experimental factors	*Paternal genetic background* (*bulls* 1*,* 2 *and* 3) *and sex* (*female and male*).
Experimental features	*Semen from three bulls sexed for X- or Y-chromosome spermatozoa were used for oocyte fertilization. Cleavage of blastomeres was monitored daily for each group, from 24* *h.p.i. until 144* *h.p.i., and results were compared between bulls and between sexes*.
Data source location	*Jaboticabal*, *Brazil*
Data accessibility	*Data is with this article*.

## Value of the data

• Kinetics of bovine embryonic development in vitro is presented (from zygote to blastocyst stage), and can be useful to compare with other systems, and dissect important time points to design experiments.

• Different bulls were used for fertilization, and the data provides separate information of each bull. This can be useful to study the effects of paternal genetic background in development.
• Data provides information on development of female and male embryos, since sexed sperm was used. This is useful to dissect time points and embryonic stages specifically affected by sex in different genetic backgrounds.

## 1 Data

We present kinetics data obtained from six time points (24, 48, 96, 120, and 144 h.p.i.), for embryos developed using semen from three different bulls. Sexed sperm was used, and groups are separated for male and female embryos.

**Fig. 1:**
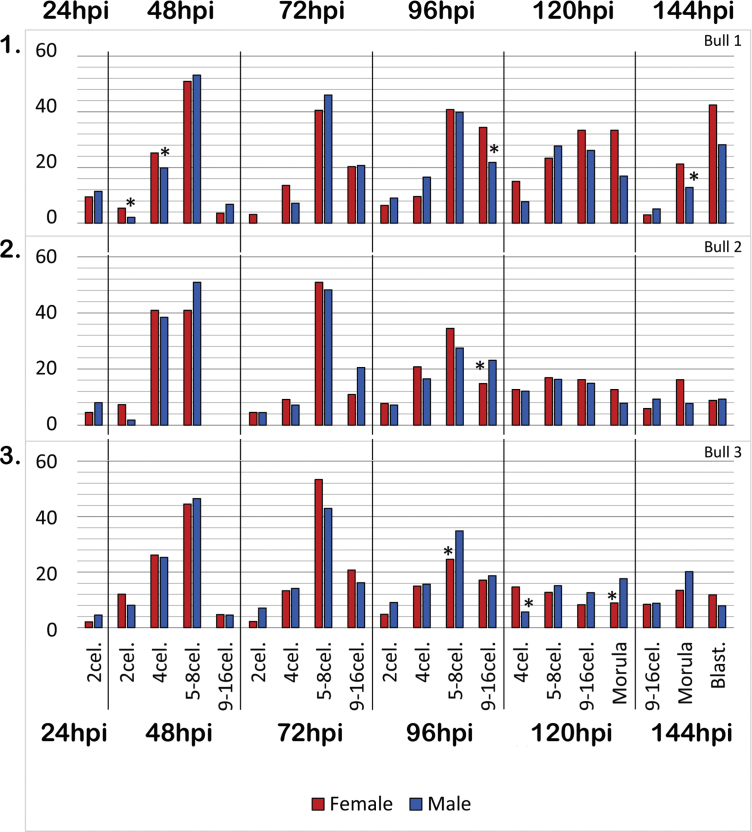
Development of male and female embryos produced after oocyte fertilization with three different bulls. Graphs show percentage of male and female embryos at main stages of development at 24, 48, 72, 96, 120 and 144 h post insemination -hpi. I. Data from presumptive zygotes produced using bull 1 semen (*n*=389 (24, 48 and 72 hpi), 385 (96 hpi), 316 (120 hpi), 233 (144 hpi)). II. Data from presumptive zygotes produced using bull 2 semen (*n*=459 (24, 48 and 72 hpi), 290 (96 hpi), 125 (120 hpi), 72 (144 hpi)). III. Data from presumptive zygotes produced using bull 3 semen (*n*=222 (24, 48 and 72 hpi), 365 (96 hpi), 283 (120 hpi), 133 (144 hpi)).

In Fig. 1, rates of representative embryonic stages at 24, 48, 72, 96, 120 and 144 h.p.i. are shown (*n*=438–1070 per timepoint), separately for female and male embryos.

Table 1 summarizes statistical analysis for grouped cleavage division׳s data. Interaction between bull and sex was significant in two cases.

**Fig. 2:**
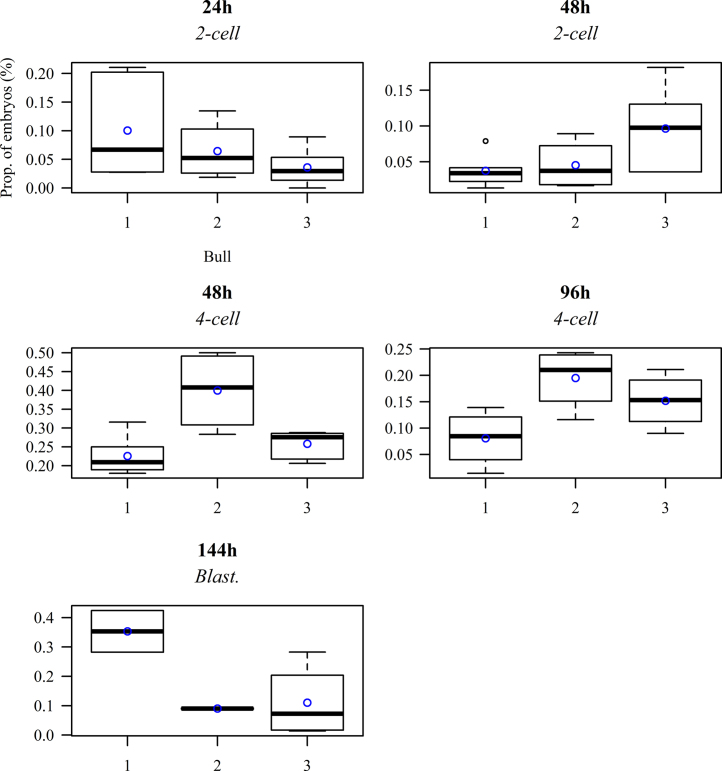
Distributions of *p* among bulls in the cases where their main effect was found to be significant, where *p* is the proportion of embryos at a specific stage of development relative to the total number of presumptive zygotes.

Fig. 2 illustrates the main significant effects of bull. At 24 h.p.i., 2-cell embryos percentage was increased in bull 1 embryos, and similar values were obtained for bulls 2 and 3 groups.

For further interpretation and discussion, see "Cell death is involved in sexual dimorphism during preimplantation development" (Oliveira et al., 2015 [1]).

## 2 Experimental design, material and methods

### 2.1 Experimental design

Matured oocytes were randomly distributed among six groups for fertilization. Semen from three bulls sexed for X- or Y-chromosome spermatozoa were used. Kinetics was assessed during development. For that, cleavage of blastomeres was monitored daily for each group, from 24 h.p.i. till 144 h.p.i., and results were compared between bulls and between sexes.

### 2.2 Supplements

Reagents and culture media were purchased from Sigma Chemical Co. (St. Louis, MO) unless otherwise stated.

### 2.3 Oocyte recovery

Bovine ovaries were collected at a local slaughterhouse and processed within 3 h after slaughter. The ovaries were washed in saline (37 °C) and follicles measuring 3–8 mm in diameter were aspirated with an 18-gauge needle coupled to a 20-mL syringe. Cumulus-oocyte complexes (COCs) presenting at least three layers of cumulus cells and homogenous cytoplasm were selected under a stereomicroscope. The COCs were washed in HEPES-buffered TCM-199 (Gibco BRL, Grand Island, NY) supplemented with 10% fetal bovine serum (FBS; Cripion, Andradina, Brazil), 16 µg/mL sodium pyruvate and 83.4 µg/mL amikacin (Instituto Biochimico, Rio de Janeiro, Brazil).

### 2.4 in vitro maturation (IVM)

Groups of 20 COCs were transferred to 100-µL drops of medium containing sodium bicarbonate-buffered TCM-199 supplemented with 10% FBS, 1.0 µg/mL FSH (Folltropin™, Bioniche Animal Health, Belleville, Canada), 50 µg/mL hCG (Profasi™, Serono, Sao Paulo, Brazil), 1.0 µg/mL estradiol, 16 µg/mL sodium pyruvate and 83.4 µg/mL amikacin, covered with sterile mineral oil (Dow Corning Co., Midland, MI) and incubated for 24 h at 38.5 °C in an atmosphere of 5% CO_2_ in air under saturated humidity.

### 2.5 in vitro fertilization (IVF)

Groups of 20 matured COCs were washed twice and transferred to 30-µL drops of TALP-IVF medium supplemented with 0.6% BSA, 10 µg/mL heparin, 18 µM penicillamine, 10 µM hypotaurine and 1.8 µM epinephrine, and covered with sterile mineral oil. Frozen-thawed straws from three different bulls, containing X-chromosome (female embryo groups) or Y-chromosome (male embryo groups) bearing spermatozoa, sorted by flow cytometry (CRV Lagoa/Sexing Technologies, Sertaozinho, Brazil) were used. For each bull, X- and Y-spermatozoa straws were obtained from the same batch of semen. Flow cytometric sperm sorting based on differences in their DNA content is the best method for separation of X- and Y-chromosome bearing spermatozoa, and its accuracy is about 90% [3], [2]. Each straw containing approximately 2 million spermatozoa was centrifuged separately on a discontinuous 45/90 Percoll gradient for 7 min at 3600×*G*. The pellet was resuspended in 700 µL TALP-IVF medium and again centrifuged for 5 min at 520×*G*. After centrifugation, 80 µL of the medium containing the pellet was collected from the bottom of the tube and homogenized in a conic tube. The final suspension was divided among five TALP-IVF drops, in a final concentration of approximately 10^4^ spermatozoa for each oocyte. The plates were incubated at 38.5 °C for 20 h in an atmosphere of 5% CO_2_ in air under saturated humidity.

### 2.6 in vitro culture (IVC)

After IVF, presumptive zygotes were partially denuded of cumulus cells by vigorous pipetting and cultured in SOF medium supplemented with 2.5% FBS and 6 mg/mL BSA at 38.5 °C in an atmosphere of 5% CO_2_ in air under saturated humidity. Remaining cumulus cells attached to plastic surface and formed a monolayer of granulosa cells. Groups of 20 presumptive zygotes were cultured in 100-µL drops, and medium was half replaced every 48 h.

### 2.7 Cleavage assessment

Embryos cultured side by side, female and male, obtained from fertilization with three different bulls were assessed daily (24, 48, 72, 96, 120 and 144 h.p.i.) for developmental progression rates.

### 2.8 Statistical analysis

All statistical analysis were performed at a 5% level of significance. Male and female groups were compared separately for each bull using Fisher׳s Exact test, in Graphpad Instat software. For this analysis, percentage of each embryonic stage was compared at each evaluated timepoint. Logistic regression models were used to analyze the effects of bull, sex and their interaction on embryonic development, separately for each evaluated time point. Problems of quasi-complete separation of data points occurred in two cases (9–16 cell at 48 h, and 2-cell at 72 h), and models were readjusted using Firth׳s penalized maximum likelihood estimation method. Only well fitted models (in terms of global likelihood-ratio Chi-Square test) were considered. Significant effects were analyzed in terms of odds ratios. SAS 9.2 software was used for this analysis.

## Figures and Tables

**Table 1:** Summary of statistical analysis.

**Time point**	**Embryonic stage**	**Global Chi-Sq. test**	**Type 3 analysis of effects**		
			**Bull**	**Sex**	**Interaction bull**^a^**sex**
24	2 cells	0.0002	0.0005	0.12	–
48	2 cells	<0.0001	0.0006	0.0072	–
48	4 cells	<0.0001	<0.0001	0.24	–
48	5–8 cells	0.12	–	–	–
48^a^	9–16 cells	0.0005	0.077	0.28	–
72^a^	2 cells	0.0016	–	–	0.042
72	4 cells	0.074	–	–	–
72	5–8 cells	0.40	–	–	–
72	9–16 cells	0.48	–	–	–
96	2 cells	0.55	–	–	–
96	4 cells	0.0015	0.0011	0.96	–
96	5–8 cells	0.33	–	–	–
96	9–16 cells	<0.0001	–	–	0.0029
120	4 cells	0.015	0.064	0.032	–
120	5–8 cells	0.73	–	–	–
120	9–16 cells	0.27	–	–	–
120	Morula	0.52	–	–	–
144	9–16 cells	0.55	–	–	–
144	Blast.	<0.0001	<0.0001	0.18	–
144	Morula	0.65	–	–	–


a In order to overcome problems of quasi-complete separation of data points, models were adjusted using Firth׳s penalized maximum likelihood estimation method, reducing bias in the parameter estimates.
